# Snail and the microRNA-200 Family Act in Opposition to Regulate Epithelial-to-Mesenchymal Transition and Germ Layer Fate Restriction in Differentiating ESCs

**DOI:** 10.1002/stem.628

**Published:** 2011-03-10

**Authors:** Jennifer G Gill, Ellen M Langer, R Coleman Lindsley, Mi Cai, Theresa L Murphy, Michael Kyba, Kenneth M Murphy

**Affiliations:** a Department of Pathology and Immunology, Washington University School of MedicineSt. Louis, Missouri, USA; b Lillehei Heart Institute and Department of Pediatrics, University of MinnesotaMinneapolis, Minnesota, USA; c Howard Hughes Medical Institute, Washington University School of MedicineSt. Louis, Missouri, USA

**Keywords:** Cell differentiation, Cell lineage, Embryonic stem cells, Mesoderm, Transcription factors, MicroRNAs

## Abstract

The reprogramming of somatic cells to inducible pluripotent stem cells requires a mesenchymal-to-epithelial transition. While differentiating ESCs can undergo the reverse process or epithelial-to-mesenchymal transition (EMT), little is known about the role of EMT in ESC differentiation and fate commitment. Here, we show that Snail homolog 1 (Snail) is expressed during ESC differentiation and is capable of inducing EMT on day 2 of ESC differentiation. Induction of EMT by Snail promotes mesoderm commitment while repressing markers of the primitive ectoderm and epiblast. Snail's impact on differentiation can be partly explained through its regulation of a number of ESC-associated microRNAs, including the microRNA-200 (miR-200) family. The miR-200 family is normally expressed in ESCs but is downregulated in a Wnt-dependent manner during EMT. Maintenance of miR-200 expression stalls differentiating ESCs at the epiblast-like stem cell (EpiSC) stage. Consistent with a role for activin in maintaining the EpiSC state, we find that inhibition of activin signaling decreases miR-200 expression and allows EMT to proceed with a bias toward neuroectoderm commitment. Furthermore, miR-200 requires activin to efficiently maintain cells at the epiblast stage. Together, these findings demonstrate that Snail and miR-200 act in opposition to regulate EMT and exit from the EpiSC stage toward induction of germ layer fates. By modulating expression levels of Snail, activin, and miR-200, we are able to control the order in which cells undergo EMT and transition out of the EpiSC state. Stem Cells 2011;29:764–776

## INTRODUCTION

Epithelial-to-mesenchymal transition (EMT) is a morphogenetic process that occurs at several stages of development, including gastrulation, neural crest migration, hair follicle morphogenesis, and in epithelial tumors during their progression toward metastasis. A number of transcription factors have been associated with controlling EMT, including Snail homolog 1 (Snai1) [1, 2], Zeb1 [3], Zeb2 [4], Twist1 [5], and Slug (Snai2) [6]. Snail has been associated with gastrulation, the earliest EMT within the developing embryo. Expression of Snail in the epiblast acts to repress E-cadherin (*Cdh1*), claudins, and occludin (*Ocln*) directly, which promote disassociation of epithelial cells to allow subsequent migration through the primitive streak. Embryos lacking Snail fail to undergo gastrulation but also exhibit a failure of allantoic-chorion fusion [7]. Snail also acts subsequently to gastrulation, as conditional deletion of Snail under control of a Meox2-Cre leads to gastrulation followed by abnormal development of mesoderm and defects in left-right asymmetry [8]. In addition, Snail has been strongly correlated with promotion of EMT and metastasis in many types of cancer [9, 10]. In *Drosophila*, Snail has also been suggested to act in fate determination, repressing neuroectoderm [11], although it is unclear whether Snail similarly influences fate choices in vertebrates.

EMT is also regulated by the microRNA-200 (miR-200) family. Forward genetic screens in tumor models and epithelial cell lines found that the five members of the miR-200 family (miR-200c, miR-141, miR-200b, miR-200a, and miR-429), located in two genomic clusters, exert repressive actions on EMT by targeting the transcription factors Zeb1 and Zeb2, which themselves repress E-cadherin [12–16]. Although no functional role of miR-200 family microRNAs (miRNAs) has been reported during early development, examination of their effects in in vitro model systems have shown that expression of miR-200 family members promote maintenance of the epithelial state, and their expression has been associated with decreased tendency of tumors to metastasize [17, 18]. In addition, expression of the miR-200 family has been examined in the chick embryo, and was excluded from mesodermal tissues [19], consistent with a proposed role in promoting nonmesenchymal cell phenotypes.

Although the miR-200 family has been shown to have a clear role in EMT, controversy exists regarding its relationship with differentiation. One study reported that miR-200 inhibits the expression of Bmi1, a polycomb repressor that acts to promote “stemness” in ESCs [20]. While this study concluded that miR-200 members promoted differentiation, others have proposed that miR-200 family members inhibit differentiation through the direct inhibition of factors like Cadherin11 and Neuropilin1 [21]. While each study supported its conclusion with specific markers, a global approach is needed to better clarify the effects of the miR-200 family on ESC differentiation. Importantly, no one has examined the effects of miR-200 on EMT or fate restriction in the ESC system.

Differentiating ESCs, which have been shown to model the gastrulating embryo, spontaneously undergo an EMT. While this has been documented [22, 23], little is known regarding the regulation or functional significance of EMT in differentiating ESCs. Because of their developmental relevance, their inherent propensity to undergo this process, and the relative ease of in vitro culture systems, ESCs serve as an ideal model system to study and better understand the genetic regulatory networks surrounding developmental EMT.

In addition to gastrulation, ESC differentiation has been shown to parallel earlier stages of embryo development. Recent studies have found that upon differentiation, murine ESCs transition from an inner cell mass-like cell to an ESC-derived epiblast-like stem cell (ESD-EpiSC) around day 2 of differentiation [24]. Cells at the latter stage maintained their pluripotency after passage in culture with activin and fibroblast growth factor (FGF). ESD-EpiSCs had similar transcriptional profiles as traditional EpiSCs, which are derived from the epithelialized mouse egg cylinder. EpiSCs are characterized by a loss of full pluripotency markers such as *Rex1*, *Dppa3*, and *Pecam1*, the maintenance of select pluripotency markers like stage-specific embryonic antigen 1 (SSEA1) and *Nanog*, and the expression of epiblast markers such as *Sox17*, *Cerberus*, and *Nodal*. The mechanisms surrounding maintenance and differentiation of the ESD-EpiSC state are currently unclear.

Interactions between Snail and the miR-200 family members have recently been suggested by studies in the reprogramming of mouse embryonic fibroblasts (MEFs) to inducible pluripotent stem (iPS) cells. First, the initiation of reprogramming of MEFs was associated with the reverse of EMT, in a process of mesenchymal-to-epithelial transition (MET) [25, 26]. A signature of MET is the induction of E-cadherin and other epithelial markers. Importantly, ectopic expression of Snail in MEFs undergoing reprogramming was associated with decreased efficiency of iPS cell generation [26]. In contrast, forced expression of E-cadherin or miR-200 family members augmented the efficiency of reprogramming [25, 27]. Although these studies did not examine the potential direct interactions of Snail and miR-200, these results showed that Snail and miR-200 families exert opposite effects during MET.

In the present study, we examined the activities and interactions of Snail and miR-200 family of miRNAs in regulating EMT and differentiation of murine ESCs. Our results indicate that induction of Snail promotes EMT and differentiation of ESCs to early mesoderm. Furthermore, Snail downregulates the expression of the miR-200 family, which is normally expressed in undifferentiated ESCs and retained in the absence of Wnt signaling. Using ESC lines that inducibly express the miR-200 family, we find that the miR-200 family acts to maintain differentiating ESCs at a state equivalent to the ESD-EpiSC, as they show increased expression of epiblast-associated genes such as *Nodal*, *Sox17*, and *Cerberus*. Furthermore, the efficient maintenance of the ESD-EpiSC stem cell state by miR-200 family members requires activin signaling. Together, we show that Snail, miR-200 family, and activin cooperate to regulate EMT and the downregulation of SSEA1 in the transition from epiblast to germ layer fate commitment in differentiating ESCs.

## MATERIALS AND METHODS

### ESC Maintenance and Differentiation

MC50 (a gift from Dr. Robert Schreiber) and modified A2lox cells [28] were maintained on feeder layers of irradiated MEFs in Iscove's modified Dulbecco's medium (IMDM) with 15% fetal calf serum (FCS), nonessential amino acids (NEAA) (0.1 mM each), L-glutamine (2 mM), sodium pyruvate (1 mM), Pen/Strep (1000 U/ml), 2-mercaptoethanol (55 μM), and leukemia inhibitory factor (LIF) (ESGRO, Chemicon International, Billerica, MA, 1,000 U/ml). Using gene-specific primers (Supporting Information Table 1), individual cDNAs were cloned from embryoid body RNA or genomic DNA and inserted into the p2lox targeting vector. To generate inducible cell lines, A2lox cells were cotransfected with gene-specific p2lox vectors and Cre recombinase as described previously [22].

Before differentiation, ESCs were passaged once in the absence of feeder layers (still in the presence of LIF). To initiate differentiation, ESCs were plated in Petri dishes in suspension at 1.5 × 10^4^ cells per milliliter in IMDM with 10% FCS, NEAA (0.1 mM each), L-glutamine (2 mM), sodium pyruvate (1 mM), Pen/Strep (1000 U/ml), and 2-mercaptoethanol (55 μM) and supplemented where indicated with Dickkopf-his (DKK-his) as described [22] or SB-431542 (10 μM, Sigma, St. Louis, MO). Gene expression was induced by addition of doxycycline (250-1,000 ng/ml, unless otherwise indicated).

### Flow Cytometry

Cells were disassociated with trypsin/EDTA for 5 minutes at 37°C and stained with antibodies. Primary antibodies are: biotin α-mE-cadherin (R&D Systems, Minneapolis, MN, 1.25 ug/ml, 1:200), PE α-Flk1 (eBioscience, San Diego, CA, Avas12a1, 1:200), APC PDGFRα (eBioscience, APA5, 1:200), and SSEA1 (DSHB, MC-480, 1:200). Secondary detection reagents are: SA-APC (BD Pharmingen, San Diego, CA, 1:400), SA-PE-Cy7 (BD Pharmingen, 1:400), and PE α-mouse IgM (BD Pharmingen, R6-60.2, 1:200). Data were acquired on a FACS Caliber (Becton Dickinson, Franklin Lakes, NJ) or FACS Canto II (Becton Dickinson) and analyzed on FlowJo (Tree Star, Ashland, OR).

### Gene Expression Analysis

To evaluate expression of individual genes, RNA was isolated with RNeasy kits (Qiagen, Germantown, MD), cDNA was synthesized using Superscript III (Invitrogen, Carlsbad, CA), and polymerase chain reaction (PCR) was performed using Taq Polymerase (Promega, Madison, WI). Nonquantitative real-time-PCR (RT-PCR) was performed using intron-spanning, gene-specific primers (Supporting Information Table 1) and cycle number varied from 25-28 cycles. Quantitative RT-PCR was performed using SYBR Green PCR Master Mix (Applied Biosystems, Carlsbad, CA) and a StepOne Plus Real Time PCR System (Applied Biosystems). Large-scale gene expression analysis was done using Affymetrix MOE430_2.0 arrays as described [29]. Data were normalized and modeled using DNA-Chip Analyzer/dChip [30, 31]. CEL Files and accompanying data were deposited in the NCBI GEO database under accession numbers GSE24289 (*A2.miR200c* data) and GSE24291 (*A2.Snail* data).

### Immunofluorescent Microscopy

Cells were differentiated as described, and at day 2, they were placed on type I collagen-coated four-well chamber slides (BD Bioscience, San Diego, CA). On day 4, cells were fixed with 2% formaldehyde in phosphate-buffered saline (PBS), blocked with 1% bovine serum albumin, 0.5% saponin in PBS, and stained with antibody in blocking solution. Primary antibodies are: biotin α-mE-cadherin (R&D Systems, 2.5 μg/ml, 1:100) and N-cadherin (BD Biosciences, 2.5 μg/ml, 1:100). Secondary antibodies are: Streptavidin-Alexa488 (Molecular Probes, Carlsbad, CA, 1:500) and Cy3 α-mIgG1 (Jackson Immunoresearch, West Grove, PA, 1:300). Nuclei were stained using Hoechst 33342 (1 μg/ml, Molecular Probes).

### miRNA Expression Analysis

To analyze expression of individual miRNAs, total RNA was isolated using *mir*Vana miRNA Isolation kit (Ambion, Austin, TX), and TaqMan microRNA assays (Applied Biosystems) were performed according to manufacture's instructions. RT-PCR was performed on a StepOnePlus Real Time PCR system (Applied Biosystems). All individual miRNA data are expressed relative to a U6 snRNA TaqMan PCR performed on the same sample. Large scale miRNA expression data was analyzed using miRNA Detection Microarrays (LC Sciences, Houston, TX).

### Inducible Expression of miRNA Family Members

miRNA families were cloned from mouse genomic DNA using primers flanking the outermost family members (Supporting Information Table 1). This DNA was then cloned into the p2lox targeting vector such that the miRNA family members would be expressed from the doxycycline-inducible promoter in the A2lox cells (see diagrams in Fig. 4A and Supporting Information Fig. 4B).

### miRNA Knockdown Studies

Fluorescein 5′-isothiocyanate–labeled miRCURY LNA miRNA Power Inhibitors (Exiqon, Woburn, MA) were obtained to inhibit miR-141, miR-200c, or nothing (negative control with no known mouse sequence homology). A2lox ESCs were plated at a density of 200,000 cells per well of a 12-well plate, and 50 nM of the indicated LNA Power Inhibitor were transfected using Lipofectamine (LF2000, Invitrogen) and OptiMEM I reduced serum medium (GIBCO, Carlsbad, CA). Media was replaced 1-day later with normal ESC media, and transfection was verified by microscopy and flow cytometry. The following day cells were harvested and set up for embryoid body differentiation as described above.

### EpiSC Culture Conditions

*A2.miR200c* ESCs were differentiated as embryoid bodies (with and without doxycycline) for 5 days. On day 5, embryoid bodies were trypsinized and subsequently cultured and passaged in EpiSC conditions in the absence of MEFs or LIF similar to methods described previously [32, 33]. After trypsinization of the embryoid bodies, cells were plated at a density of 115,000 cells per well of a six-well plate (precoated with FCS overnight and washed with PBS). Cells were cultured in IMDM with 20% Knockout Serum Replacement, NEAA (0.1 mM each), L-glutamine (2 mM), sodium pyruvate (1 mM), Pen/Strep (1000 U/ml), 2-mercaptoethanol (55 μM), 5 ng/mL FGFb (GIBCO), and 20 ng/mL rh-activin A (GIBCO). Media was replenished daily. To passage, colonies were removed with a cell scraper, triturated into small clusters with a P200 pipette tip, and passaged every 2-3 days with typically a 1:3 split.

## RESULTS

### Snail Promotes EMT and Early Mesoderm Commitment in Day 2 Differentiating ESCs

In a previous study, we examined the induction of mesoderm in differentiating ESCs by several transcription factors including mesoderm posterior 1 (Mesp1) [29]. Mesp1 also induced EMT in differentiating ESCs, which appeared to be because of the intermediate induction of Snail, a factor strongly induced by Mesp1 and which we showed was able to independently downregulate E-cadherin expression. We decided to use this system to better understand the effects of EMT on normal ESC differentiation.

**Figure 1:**
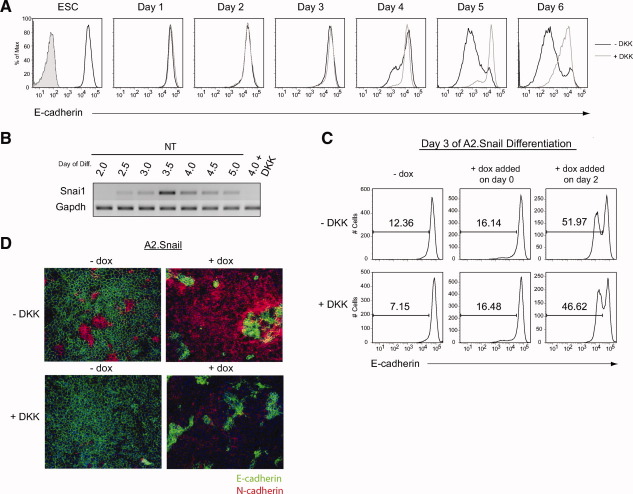
Snail promotes epithelial-to-mesenchymal transition in day 2 differentiating ESCs. **(A):** E-cadherin protein expression during MC50 ESC differentiation was analyzed by flow cytometry in serum-containing differentiation conditions without (black line) or with (gray line) addition of the Wnt inhibitor DKK at day 0. A secondary antibody control is shown as a shaded plot in the ESC panel. **(B):** *Snai1* and *Gapdh* expression were measured on days 2, 3, 4, and 5 of normal MC50 ESC differentiation by real-time-polymerase chain reaction. Expression levels were also examined on day 4 of MC50 ESC differentiation after treatment with DKK from day 0. **(C):** FACS histogram of E-cadherin expression on day 3 of ESC differentiation. *A2.Snail* ESCs were differentiated with or without DKK from day 0 and with or without addition of doxycycline at day 0 or day 2. **(D):** *A2.Snail* ESCs were differentiated with or without DKK and doxycycline as outlined in Supporting Information Figure 1A. Cells were placed on type I collagen-coated four-well chamber slides on day 2 and fixed and stained on day 4. E-cadherin is shown in green and N-cadherin is shown in red. Abbreviations: DKK, Dickkopf; dox, doxycycline; NT, no treatment.

To characterize the timecourse of EMT during ESC differentiation, we performed a FACS analysis of E-cadherin expression on differentiating MC50 ESCs. E-cadherin is normally maintained for at least 3 days following withdrawal of LIF, but is increasingly lost on days 4, 5, and 6 in a Wnt-dependent manner (Fig. 1A). The expression of Snail in differentiating ESCs closely corresponds to the loss of E-cadherin, showing a peak of expression on day 3.5 immediately preceding the onset of E-cadherin downregulation (Fig. 1B). Snail expression is absent in DKK-treated cultures, which we have previously shown maintain E-cadherin expression and fail to undergo EMT (Fig. 1A) [22]. Using an ESC line with doxycycline-inducible Snail (*A2.Snail*), we next sought to characterize how Snail affected E-cadherin levels in differentiating ESCs. We examined cultures treated with and without DKK, and with doxycycline added on day 0 or just before its normal endogenous expression on day 2. Interestingly, Snail was capable of downregulating E-cadherin when induced on day 2, but not in day 0 cultures (Fig. 1C), suggesting that ESCs may lack factors required for Snail's function. In adherent differentiation cultures, induction of Snail on day 2 led to dramatic changes in morphology, where Snail-induced cells acquired spindle-like morphologies and adhered as single cells (Supporting Information Fig. 1A, 1B).

To further investigate EMT after Snail induction, we induced Snail on day 2, in the presence or absence of DKK (Supporting Information Fig. 1A). RT-PCR showed that expression of E-cadherin is still evident on day 3 of differentiation in cultures without Snail induction, but is repressed by induction of Snail following doxycycline treatment (Supporting Information Fig. 1C). Correspondingly, the mesenchymal marker fibronectin is induced by Snail in this system. Although mesenchymal differentiation is normally blocked by inhibition of Wnt signaling, Snail is capable of powerful upregulation of fibronectin expression (Supporting Information Fig. 1C).

In addition, Snail induces a switch from E-cadherin expression to N-cadherin expression (Fig. 1D) in the majority of differentiating ESCs. The upregulation of N-cadherin is more robust in the presence of Wnt signaling, but it also occurs in DKK cultures. These results show that Snail's actions in this differentiating ESC culture system promote features of an EMT.

**Figure 2:**
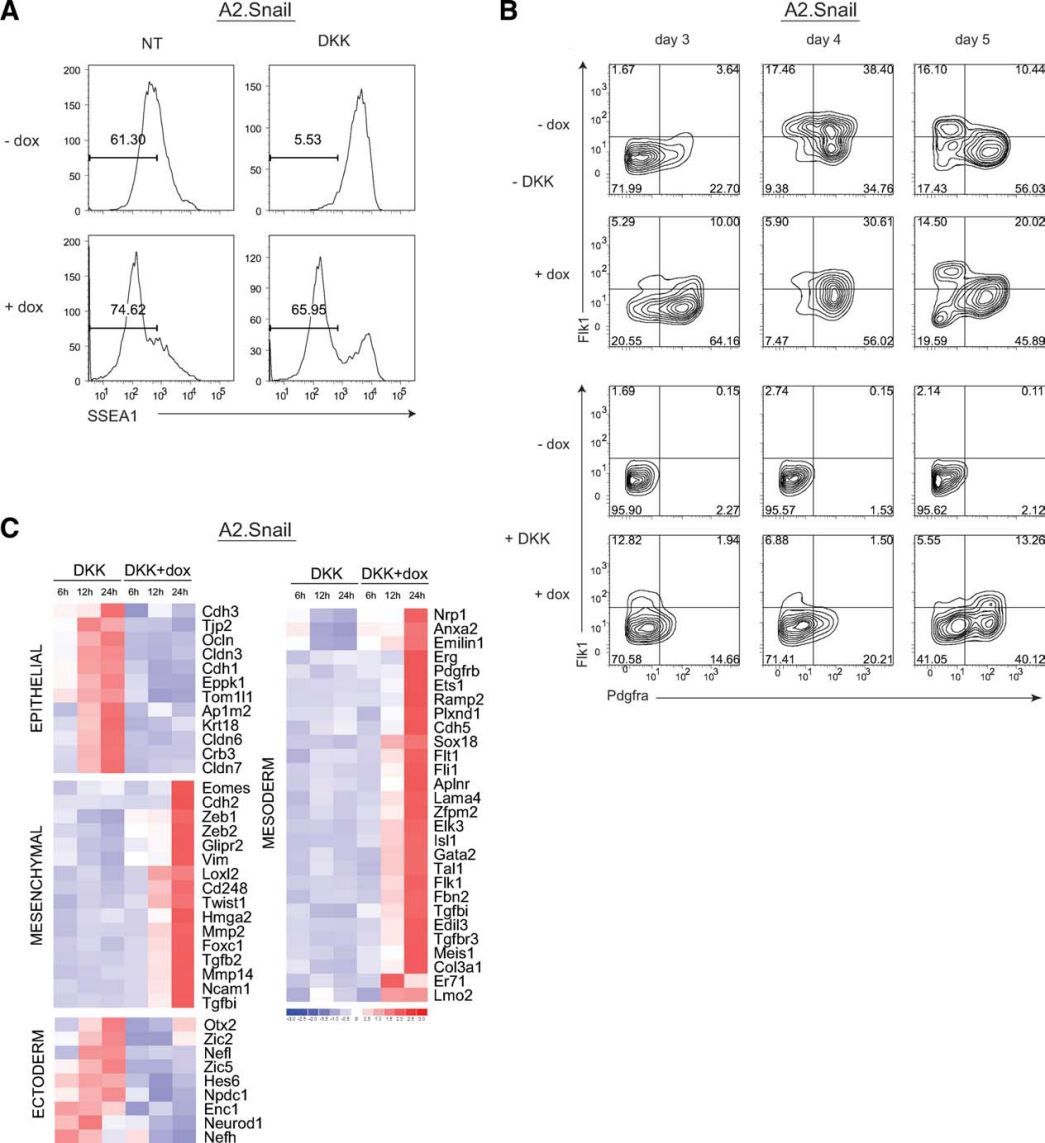
Snail accelerates ESC differentiation while promoting mesoderm differentiation. **(A):** FACS analysis of SSEA1 protein expression on day 4 of *A2.Snail* differentiation in the presence or absence of doxycycline at day 2 and DKK added at day 0. **(B):** FACS analysis of Flk1 and PDGFRα protein expression on days 3, 4, and 5 of ESC differentiation. *A2.Snail* cells were differentiated as in Supporting Information Figure 1A. **(C):** Relative gene expression in *A2.Snail* ESCs differentiated with DKK in the absence or presence of doxycycline. RNA was collected at 6, 12, and 24 hours after doxycycline addition (corresponding to days 2.25, 2.5, and 3 of differentiation). Red and blue indicate increased and decreased expression, respectively. Raw data can be found in the NCBI GEO database under accession number GSE24291. Abbreviations: DKK, Dickkopf; dox, doxycycline; NT, no treatment.

Because a number of pluripotency transcription factors including Sox2 and Oct4 have been found to repress Snail [26], we wondered whether Snail may reciprocally play a role in limiting ESC pluripotency. To examine potential actions of Snail on differentiation, we first examined the ability of Snail to regulate expression of the pluripotency marker SSEA1 (Fig. 2A). Normally, SSEA1 expression is gradually downregulated during differentiation of ESCs in vitro. Interestingly, we find that the loss of SSEA1 by differentiating ESCs is blocked by the addition of the Wnt inhibitor DKK. Induction of Snail in differentiating ESCs significantly accelerates the loss of SSEA1. Moreover, even in the presence of DKK, induction of Snail causes the downregulation of SSEA1 in the majority of ESCs. Through a titration of doxycycline, we can confirm that this effect of Snail can be achieved at levels similar to endogenous and in a dose-dependent manner (Supporting Information Fig. 2). In addition to SSEA1 downregulation, we also show that Snail downregulates expression of Sox2 and Oct4 transcripts, but not Nanog, by day 4 of differentiation in DKK + dox cultures (Supporting Information Fig. 3). Thus, Snail promotes the loss of pluripotency markers of ESCs, suggesting a potential role for Snail in promoting differentiation.

To refine this analysis and determine whether Snail-induced loss of pluripotency promoted fate determination, we examined two markers of mesodermal fate, Pdgfrα, and Flk1 (Fig. 2B). Normal differentiating ESCs undergo EMT and begin to express these markers of mesodermal fates beginning on day 3 and continuing through day 5. Induction of Snail in differentiating ESCs accelerated the progression of Pdgfrα expression and Flk1 expression (Fig. 2B, upper panels). Importantly, even when mesoderm differentiation was blocked through the addition of the Wnt signaling inhibitor DKK, the induction of Snail was still able to induce expression of these markers of mesoderm (Fig. 2B, lower panels).

To obtain a global view of the effects of Snail on ESC differentiation, we carried out microarrays of differentiating *A2.Snail* ESCs under conditions of Wnt signaling inhibition to prevent endogenous-promoted EMT and mesoderm differentiation (Fig. 2C). In the absence of Snail induction, these conditions lead to a pattern of differentiation that is strongly skewed toward an epithelial phenotype and ectodermal fates (Fig. 2C and [22]). However, upon induction of Snail, expression of epithelial and ectodermal markers are inhibited (e.g., *Otx2*, *Zic5*, and *Cldn6*), while genes associated with mesenchymal and mesodermal fates are strongly induced (e.g., *Mmp2*, *Ncam1*, *Isl1*, and *Gata2*). These results establish a precedent for the ability of Snail to alter patterns of cell type differentiation during ESC differentiation and indicate that Snail expression favors mesodermal rather than ectodermal fate choices.

### Snail Regulates miR-200 Family Member Expression to Promote EMT and ESC Differentiation

**Figure 3:**
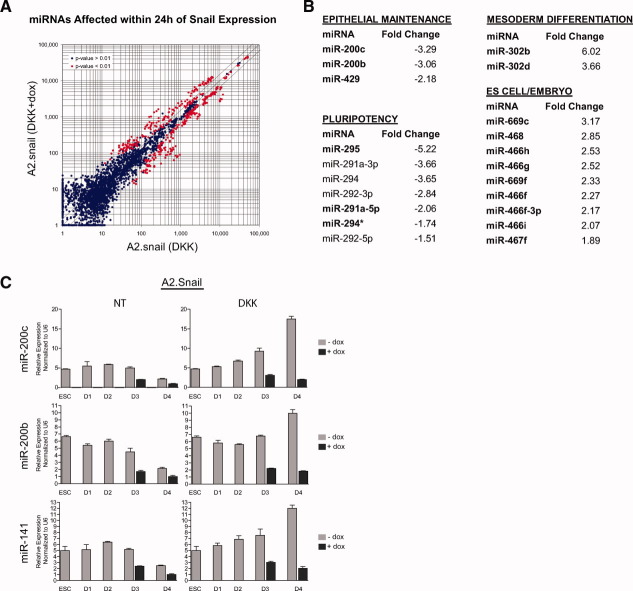
Snail represses the expression of the microRNA-200 family and alters expression of multiple ESC microRNA (miRNA) families. **(A):** Plotted are relative expression of 627 miRNAs in DKK and DKK + dox conditions on day 3 of *A2.Snail* ESC differentiation as described in Supporting Information Figure 1A. Red dots represent miRNAs, which were considered to be differentially expressed in the two samples with a *p* < .01. **(B):** Table of miRNA families significantly altered within 24 hours of *Snail* expression. miRNAs in bold have been validated by an additional miRNA chip (biological replicate) and/or TaqMan miRNA quantitative polymerase chain reaction (PCR) assays. **(C):** Real-time PCR of selected miRNAs using TaqMan miRNA assays. RNA samples were collected daily from ESCs differentiated as described in Supporting Information Figure 1A. Doxycycline treatment is indicated by light gray bars (no doxycycline) and dark gray bars (doxycycline). Samples were normalized to U6 snRNA. Expression for each miRNA is shown relative to the condition with the lowest miRNA expression (day 4, NT + dox), which was set at 1. Abbreviations: DKK, Dickkopf; dox, doxycycline; miRNA, microRNA; NT, no treatment.

Snail reportedly acts in the manner of a transcriptional repressor, and yet some of its actions involve strong induction of target genes. Therefore, we wondered whether Snail might regulate the expression of miRNAs, which could serve to repress targets of Snail that may be indirectly induced. To address this, we carried out a global screen for miRNA expression under conditions of variable Snail expression (DKK and DKK + dox) (Fig. 3A). Among the miRNAs that were significantly repressed by induction of Snail in ESCs were members of the miR-200 family (Fig. 3B and Supporting Information Table 2). In addition, several miRNAs that have been associated with maintenance of pluripotency in ESCs were also inhibited by the induction of Snail. Notably, one family of miRNAs, miR-302, that is associated with differentiated mesoderm, was induced by Snail (Fig. 3B).

As the miR-200 family has been associated with EMT through its targeted repression of Zeb1 and Zeb2, we wanted to more closely examine the expression pattern and relationship with Snail induction during ESC differentiation (Fig. 3C). During normal ESC differentiation (without inhibition of Wnt signaling), miR-200 family members are maintained until approximately day 3 of differentiation, and decreased by approximately 50% on day 4. Upon induction of Snail at day 2 of differentiation, miR-200 family members are reduced by 50% after 24 hours and further reduced by day 4, indicating that Snail inhibits expression of each of the miR-200 family members examined. Furthermore, we observe that the expression of the miR-200 family members is strongly augmented under conditions that promote neuroectodermal fate and prevent EMT (Fig. 3C). In particular, the addition of the Wnt inhibitor DKK blocks mesoderm differentiation [22] and causes the gradual accumulation of higher levels of miR-200c, miR-200b, and miR-141 (Fig. 3C). Importantly, even under these conditions in which high levels of miR-200 are normally expressed, the induction of Snail strongly represses the expression of endogenous miR-200 family miRNAs. These results indicate that Snail acts to repress the expression of the miR-200 family.

**Figure 4:**
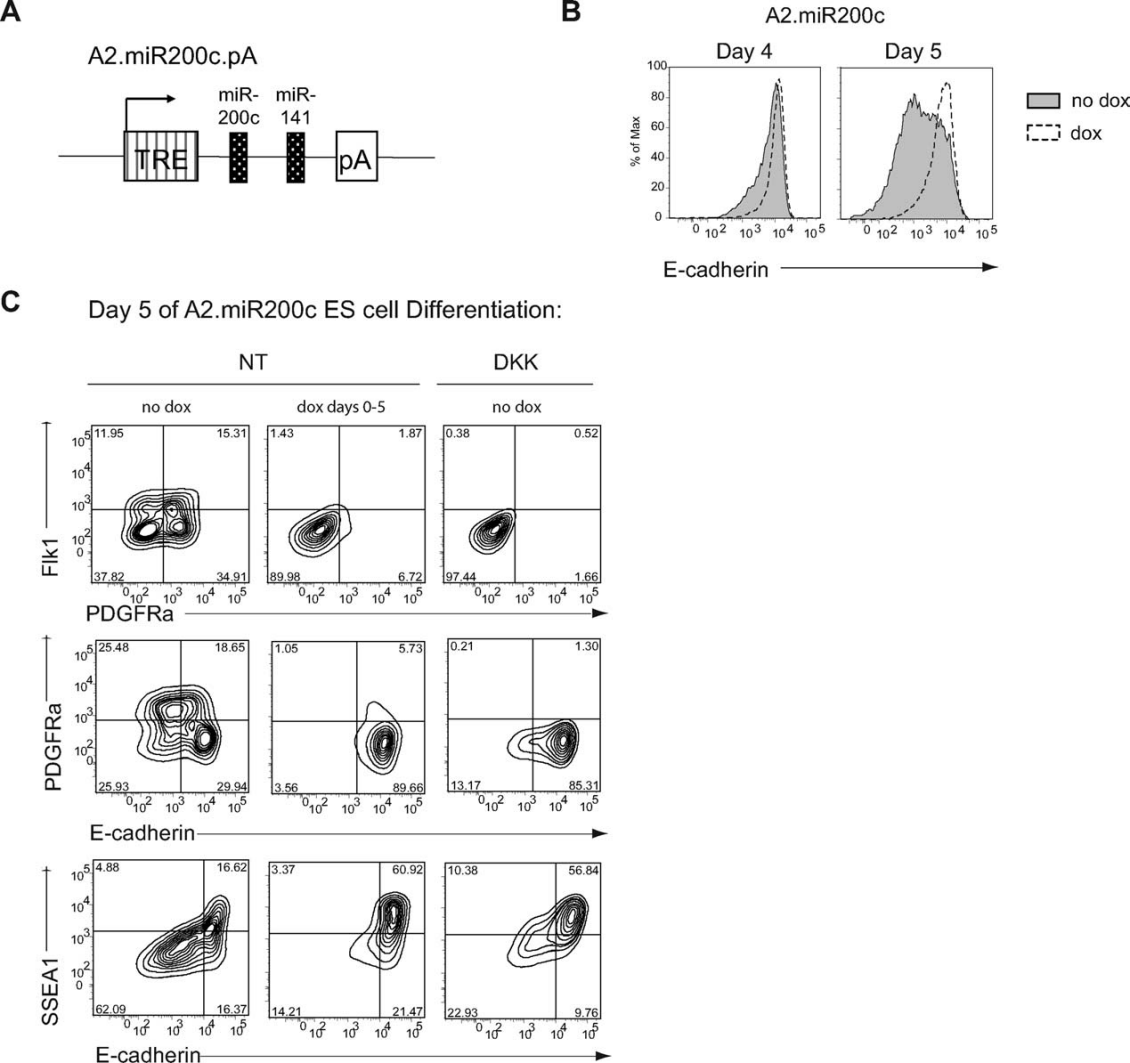
The microRNA-200 family inhibits epithelial-to-mesenchymal transition and mesoderm formation in differentiating ESCs. **(A):** Diagram of the tetracycline-inducible locus for the *A2.miR200c* ESC line. **(B):** FACS analysis of E-cadherin on days 4 and 5 of *A2.miR200c* ESCs differentiated as described in Supporting Information Figure 1A. Shown are conditions with and without the addition of doxycycline. Shown are gated live cells. **(C):** FACS analysis of Flk1, PDGFRα, E-cadherin, and SSEA1 on day 5 of *A2.miR200c* ESC differentiation. Doxycycline was added beginning on day 0 and replenished every other day. Shown are gated live cells. Abbreviations: DKK, Dickkopf; dox, doxycycline; Flk1, fetal liver kinase 1; miR-200c, microRNA-200c; miR-141, microRNA-141; NT, no treatment; pA, polyA; PDGFRα, platelet-derived growth factor receptor α; SSEA1, stage-specific embryonic antigen 1; TRE, tetracyclin response element.

Given its expression pattern in ESC differentiation and in response to Snail, we wanted to determine whether the miR-200 family could prevent EMT in ESC differentiation as it has been shown to do in tumor models. Therefore, we generated ESC lines harboring inducible expression of miR-200c and miR-141 genomic cluster (*A2.miR200c*). In this cell line, miR-200c/141 is expressed downstream of a doxycycline-inducible transcriptional element (Fig. 4A and Supporting Information Fig. 4A). Without induction of miR-200c/141, ESCs normally begin to lose expression of E-cadherin by day 4 after induction of differentiation, with a majority of ESCs having lost E-cadherin on day 5 (Fig. 4B). However, when miR-200c/141 is induced, differentiating ESCs maintain expression of E-cadherin at day 4 and day 5 opposite to the effects of Snail on E-cadherin expression (compare with Fig. 1C, 1D).

In addition to examining effects on EMT, we wanted to further understand how prevention of EMT by the miR-200 family might affect ESC differentiation. We first examined the effects of miR-200c/141 on the expression of mesodermal markers and pluripotency markers in differentiating ESCs (Fig. 4C). Normal differentiating ESCs have induced the mesoderm markers Pdgfrα and Flk1 in a substantial number of cells by day 5 after differentiation (Fig. 4C, first column, upper panel). However, by maintaining expression of miR-200c/141 during ESC differentiation, these mesodermal markers failed to be induced (Fig. 4C second column, upper panel). Here, the level of expression of Flk1 and Pdgfrα in the presence of miR-200c expression is nearly as low as when mesoderm differentiation is blocked through the addition of the Wnt inhibitor DKK (Fig. 4C, third column, upper panel). In addition, we examined the effect of miR-200c on the pluripotency marker SSEA1 (Fig. 4C, bottom). Normally, by day 5 of ESC differentiation, approximately 85% of ESCs have lost SSEA1 expression (i.e., 62 + 16%) and have significantly down regulated E-cadherin expression. However, when miR-200c expression is maintained, SSEA1 expression is retained on the majority of ESCs with only 36% becoming SSEA1 negative. In addition, many fewer ESCs lose expression of E-cadherin when miR-200c expression is maintained. These results indicated that maintenance of miR-200c/141 expression during ESC differentiation acts to prevent loss of E-cadherin, to prevent induction of mesodermal markers, and to prevent the downregulation of SSEA1. We found similar results when we generated an ESC line with an inducible miR-200b/200a/429 cluster (Supporting Information Fig. 4B–4D). Furthermore, robust induction of a different miRNA (miR-335) failed to have the same effects as the miR-200 family, suggesting specificity (Supporting Information Fig. 5). The effects of the miR-200 family are opposite to those of Snail shown above.

To determine whether loss of miR-200c/141 is sufficient for E-cadherin and SSEA1 downregulation, we used LNA miRNA inhibitors (Exiqon) for loss-of-function studies. ESCs transfected with anti-miR-141 or anti-miR-200c showed a slightly increased percentage of cells with downregulated E-cadherin and SSEA1 on days 4 and 5 of differentiation when compared with a negative control inhibitor (Supporting Information Fig. 6A, 6B). Because of the existing redundancy and target overlap in the miR-200 family, we hypothesize that the remaining uninhibited family members (miR-200a, miR-200b, and miR-429) may still be functioning to limit EMT and differentiation. Inhibition of miR-200c/141 alone was not sufficient to promote mesoderm induction (Supporting Information Fig. 6C).

### Expression of miR-200 Family miRNAs Promotes Maintenance of the ESD-EpiSC State

**Figure 5:**
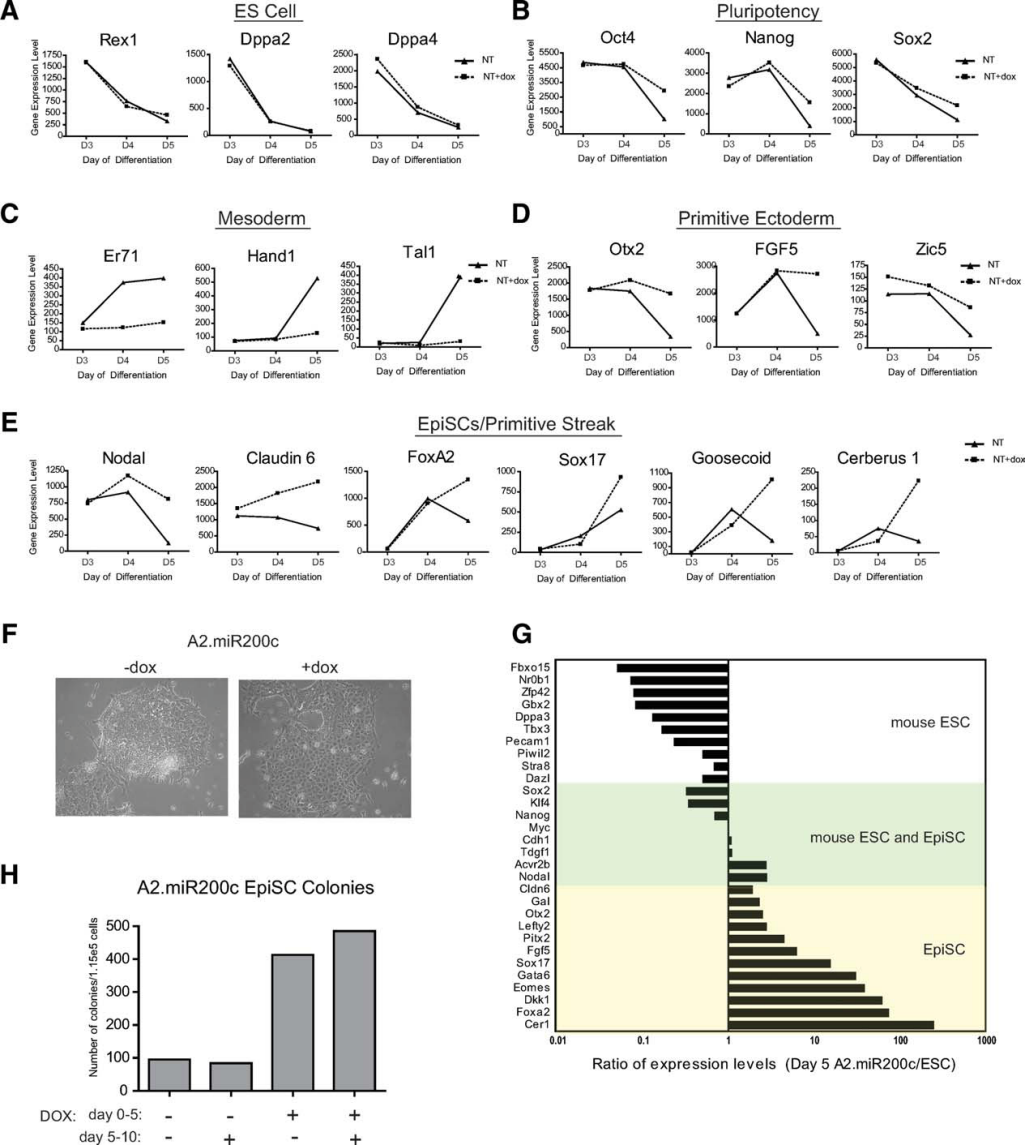
The maintenance of microRNA-200 expression generates cells with an epiblast-like stem cell (EpiSC) transcriptional profile and phenotype. Relative gene expression in *A2.miR200c* ESCs differentiated in the absence or presence of doxycycline from day 2 and replenished on day 4. RNA was collected on days 3, 4, and 5 of ESC differentiation for microarray analysis. Raw data can be found in the NCBI GEO database under accession number GSE24289. **(A–E):** Shown are relative gene expression levels of the indicated markers over the timecourse of *A2.miR200c* differentiation with (dashed line) and without (filled line) doxycycline. Relative gene expression levels of markers of ESCs **(A)**, pluripotency **(B)**, mesoderm **(C)**, primitive ectoderm **(D)**, and EpiSCs/primitive streak **(E)**. **(F):** Light microscopy of day 3.5 adherent ESC differentiation of *A2.miR200c* cells with and without doxycycline addition on day 2. **(G):** Microarray data from day 5 *A2.miR200c* ESCs treated with doxycycline were normalized to MC50 ESC microarray expression data. Ratios of selected genes were plotted on a log scale. **(H):** *A2.miR200c* ESCs were differentiated for 5 days as embryoid bodies (with and without doxycycline). On day 5, cells were trypsinized and replated in EpiSC culture conditions (see Materials and Methods and Supporting Information Fig. 7 for further details), with and without doxycycline maintenance. On day 10, the number of colonies per well were counted and documented. Abbreviations: dox, doxycycline; EpiSC, epiblast-like stem cell; NT, no treatment.

Because we observed that maintenance of miR-200c/141 inhibited expression of mesodermal markers while maintaining expression of SSEA1, we wondered whether the miR-200 family acted simply by preventing all differentiation of ESCs. To address this question, we carried out global gene expression analysis of ESCs at days 3, 4, and 5 of differentiation either in the presence or absence of miR-200c/141 expression from day 2. First, the induction of miR-200c and miR-141 did not simply prevent all changes associated with ESC differentiation. Differentiating ESCs rapidly downregulate the ESC markers *Rex1*, *Dppa2*, and *Dppa4*, and the loss of these markers was not altered upon induction of miR-200c/141 (Fig. 5A). The pluripotency markers *Oct4*, *Nanog*, and *Sox2* are also downregulated during normal ESC differentiation, but induction of miR-200c and miR-141 led to only a partial loss of these genes (Fig. 5B).

Consistent with our findings that miR-200c/141 inhibited Pdgfrα and Flk1 expression (Fig. 4C), we found that mesoderm-associated genes were strongly blocked by miR-200c and miR-141. For example, *Er71*, a gene associated with blood and endothelium, and *Tal1*, a gene associated with hemogenic endothelium, are normally strongly induced in differentiating ESCs, but their expression was virtually extinguished by miR-200c/141 (Fig. 5C). In contrast, genes associated with primitive ectoderm (e.g., *Otx2*, *Fgf5*, and *Zic5*) were maintained by expression of miR-200c/141 (Fig. 5D). Interestingly, when we looked for other genes strongly altered upon induction of miR-200c and miR-141, we identified a number of genes that have recently been associated with expression in EpiSCs and ESD-EpiSCs (Fig. 5E). For example, *Nodal*, *Claudin6* (*Cldn6*), and *Cerberus 1* (*Cer1*) are genes associated with the EpiSC and are substantially elevated upon expression of miR-200c/141. As EpiSCs have also been shown to maintain primitive ectoderm markers, these findings are consistent with the notion that maintenance of miR-200c/141 expression causes a failure of cells to progress past the EpiSC stage of ESC differentiation.

Another feature associated with EpiSCs is in the nature of its morphology during culture. We find that a cell line harboring inducible expression of miR-200c/141 exhibits a phenotype that can be switched upon induction of expression by doxycycline. In the absence of induction, differentiating ESCs in adherent cultures grow as a round cluster of mostly spindle-shaped cells attached to each other. In contrast, induction of miR-200c/141 causes the morphology to switch to a homogenous flattened monolayer of cells tightly bound to the culture substrate (Fig. 5F).

To better determine how similar miR-200c/141-induced cultures were to EpiSCs, we examined the expression profile of a number of genes characterized as being mouse ESC-specific, mouse EpiSC-specific, and genes shared between the two subsets [34]. In comparing day 5 miR-200c/141-induced cultures to undifferentiated ESCs, we see that like EpiSCs, miR-200c/141 induced cultures have downregulated a number of ESC-specific transcripts while partially maintaining other markers such as *Nanog* and *Nodal*. As seen in EpiSCs, *Cer1*, *Foxa2*, and other targets of nodal/activin signaling were significantly upregulated.

Because EpiSCs can be maintained through repeated passages in FGF and activin (in the absence of LIF), we wanted to determine whether *A2.miR200c* doxycycline-treated cells would also share this property with EpiSCs. To test this, we treated *A2.miR200c* ESCs with or without doxycycline from day 0 to day 5 of differentiation (Supporting Information Fig. 7A, B). On day 5, we trypsinized embryoid bodies and transferred cells into FGF + activin EpiSC culture conditions. Relative to untreated cultures, samples derived from day 5 doxycycline-treated cultures generated over fourfold more colonies after culture in EpiSC culture conditions from day 5 to day 10 (Fig. 5H). In addition to generating more colonies, these colonies were notably larger and had a more flattened, EpiSC-like appearance (Supporting Information Fig. 7A). Within a few passages, the fewer and smaller colonies derived from day 5 untreated embryoid body cultures differentiated and could not be sustainably cultured. In contrast, the colonies originally derived from day 5 doxycycline-treated cultures continued to grow and maintain EpiSC-like morphology in FGF and activin (Supporting Information Fig. 7A). After 50 days in EpiSC culture (and nearly 20 passages), we isolated RNA from these cells to determine whether they maintained markers of pluripotency and EpiSCs. As assessed by quantitative RT-PCR, these colonies continued to express pluripotency markers found in both ESCs and EpiSCs (Nanog and Oct4) as well as markers more specific to EpiSCs (FGF5 and nodal) (Supporting Information Fig. 7C). The cultures that were consistently maintained in doxycycline had higher expression levels of both pluripotency and EpiSC markers, suggesting that doxycycline-induced miR-200c/141 expression may also assist in maintaining these cells as EpiSCs throughout long-term passaging.

In summary, gene expression and morphology suggest that the maintenance of expression of miR-200 family miRNAs allows differentiation with loss of several ESC markers, but promotes maintenance of the EpiSC state while preventing further differentiation toward subsequent differentiated states such as mesoderm.

### Inhibition of Activin Promotes EMT and Downregulation of the miR-200 Family Concomitant with Neuroectoderm Differentiation

EpiSCs and ESD-EpiSCs are maintained through the actions of activin [24, 32, 34], a TGFβ family member expressed throughout the anterior primitive streak in the developing embryo. It has previously been shown that removal of activin from ESC cultures promotes the differentiation of cells toward neuroectodermal lineages [35]. However, the role of miRNAs and EMT in this process has not been examined.

**Figure 6:**
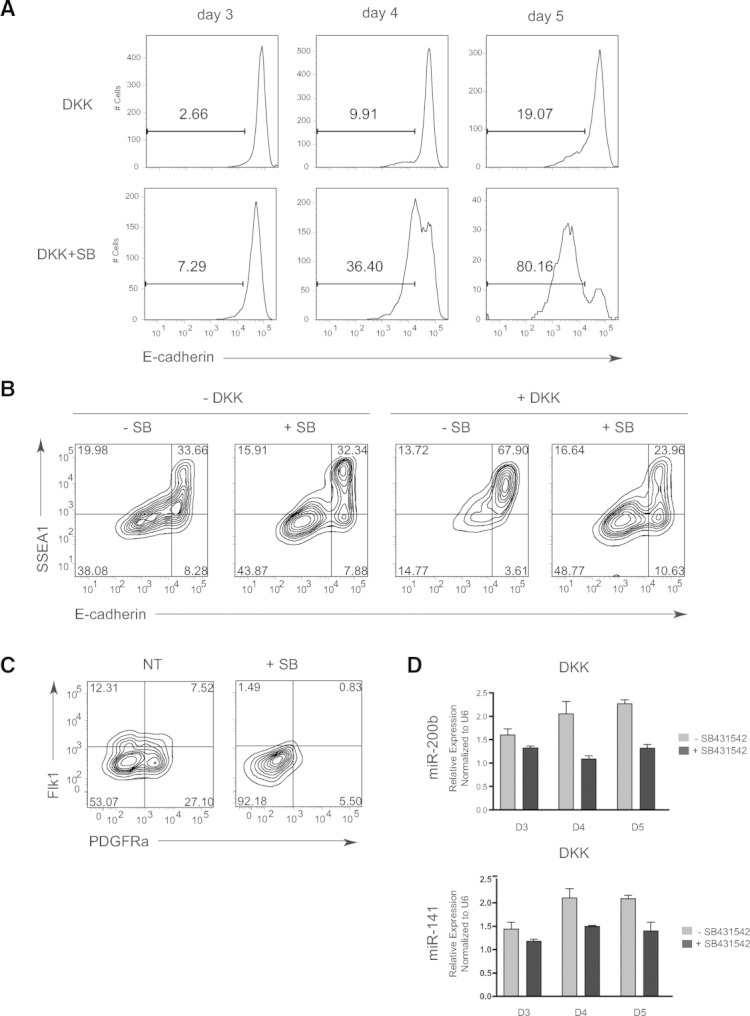
Inhibition of activin promotes epithelial-to-mesenchymal transition and differentiation while downregulating the microRNA-200 (miR-200) family. **(A):** *A2.Snail* ESCs were differentiated as described in Supporting Information Figure 1A in the presence of DKK with or without the addition of 10 μM SB-431542. Live cells were gated and a histogram of E-cadherin expression on days 3, 4, and 5 of differentiation was plotted. **(B):** FACS plots illustrating E-cadherin and SSEA1 expression on day 5 of ESC differentiation as described in **(A)**. **(C):** FACS plots of Flk1-PE and PDGFRα-APC in day 5 ESCs treated with or without 10 μM SB-431542. **(D):** Real-time polymerase chain reaction of miR-200b and miR-141 microRNAs (miRNA) using TaqMan miRNA assays. RNA samples were collected daily from ESCs differentiated with DKK from day 0, with or without the addition of SB-431542. Samples were normalized to U6 snRNA. Abbreviations: DKK, Dickkopf; Flk1, fetal liver kinase 1; miR-200b, microRNA-200b; miR-141, microRNA-141; NT, no treatment; PDGFRα, platelet-derived growth factor receptor α; SB, SB-431542; SSEA1, stage-specific embryonic antigen 1.

We hypothesized that inhibition of activin with SB-431542, a small molecule inhibitor of ALK-4, ALK-5, and ALK-7, would prevent maintenance of the ESD-EpiSC in differentiating ESC cultures and, therefore, lead to EMT and downregulation of the miR-200 family. When we treated differentiating ESC cultures with SB-431542 on day 2, we saw downregulation of E-cadherin as well as SSEA1 in both the presence and absence of DKK (Fig. 6A, 6B and Supporting Information Fig. 8). Consistent with SB-431542 promoting neuroectoderm, we see a loss of the early mesoderm markers Flk1 and Pdgfrα (Fig. 6C).

Along with EMT, inhibition of activin also led to the downregulation of miR-200b and miR-141 (Fig. 6D), consistent with the role of these miRNAs in maintaining cells in an epithelial, EpiSC-like state. Therefore, both Snail and activin inhibition are capable of inducing EMT and promoting exit from the epiblast as assessed by SSEA1 downregulation. However, the germ layer induction differs in these two scenarios as Snail promotes mesoderm fates while SB-431542 biases toward neuroectoderm.

### Snail and the miR-200 Family Cooperate with Activin to Regulate Exit from the ESD-EpiSC State

**Figure 7:**
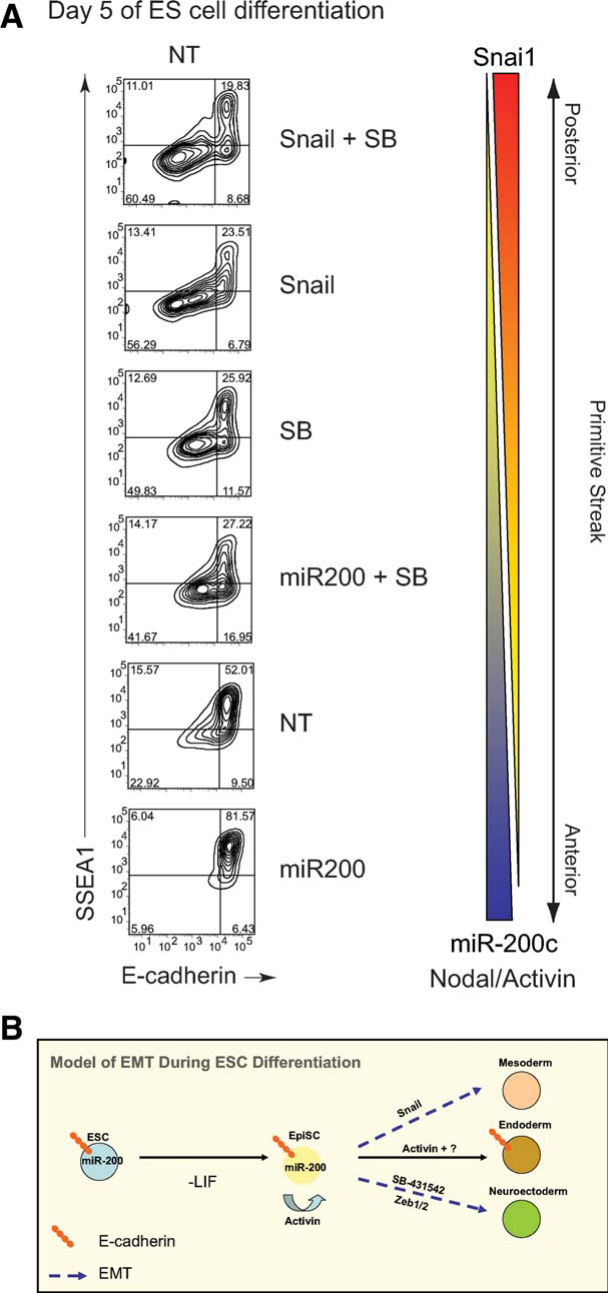
Snail and microRNA-200 cooperate with activin to regulate exit from the ESC-derived epiblast-like stem cell state. **(A):** *A2.miR200c* and *A2.Snail* cells were differentiated as described in Supporting Information Figure 1A, with or without the addition of 10 μM SB-431542 and with or without doxycycline on day 2 (replenished on day 4 for *A2.miR200c*). Shown is the FACS analysis for SSEA1 and E-cadherin on day 5 of differentiation. Unless indicated by Snail expression, all cell lines shown were *A2.miR200c*. **(B):** Proposed model of epithelial-to-mesenchymal transition during ESC differentiation. Abbreviations: EMT, epithelial-to-mesenchymal transition; EpiSC, epiblast-like stem cell; LIF, leukemia inhibitory factor; miR-200, microRNA-200; NT, no treatment; SB, SB-431542; SSEA1, stage-specific embryonic antigen 1.

Because activin appeared to be important in preventing EMT and differentiation in both no treatment and DKK-treated cultures, we wanted to know whether the miR-200 family required activin for maintenance of cells in an SSEA1+/E-cadherin+ ESD-EpiSC state. Therefore, we examined differentiation of ESCs harboring inducible miR-200c/141 in the presence or absence of SB-431542 from day 2. In the absence of SB-431542, miR-200/141 was capable of retaining cells as E-cadherin+/SSEA1+ (Fig. 7A, bottom two plots). Importantly, when activin signaling is inhibited by SB-431542, miR-200 is no longer capable of preventing cells from differentiating or undergoing EMT (Fig. 7A, fourth and sixth plots). This may be in part due to the miR-200 family's inability to maintain sufficiently low levels of the transcription factors Zeb1 and Zeb2 (Supporting Information Fig. 9). Furthermore, when activin is inhibited in cultures expressing Snail, more cells undergo EMT and differentiation than in either condition alone (Fig. 7A, top three rows). Considering the expression pattern of these factors in the gastrulating embryo, a temporal order of migration and differentiation of cells from the epiblast through the primitive streak is apparent. Snail is expressed at highest levels in the posterior primitive streak, while nodal/activin signaling is highest in the anterior streak. Our finding that miR-200 is excluded from mesoderm in differentiating ESCs corresponds with expression data in the chick [19], indicating that miR-200 has an inverse expression pattern to Snail and is correlated with activin expression in the gastrulating embryo. By modulating expression levels of Snail, activin, and miR-200, we are able to control the order in which cells undergo EMT and transition out of the ESD-EpiSC state. These findings correlate with the mouse embryo where cells of the posterior streak are the first to migrate, followed by a subsequent cascade of migration by cells positioned more anterior. Taken together, we find that Snail, activin, and miR-200 family cooperate to regulate epiblast differentiation and progression through EMT and germ layer fate commitment.

## DISCUSSION

Snail has been associated with E-cadherin repression during gastrulation, but its downstream pathways and association with fate determination have not been well-characterized. In this study, we have uncovered a role for Snail in promoting EMT and mesoderm differentiation within a specific timeframe of ESC differentiation corresponding to the early epiblast. During this time, Snail alters the expression of a number of miRNAs, including the miR-200 family. Further, we have demonstrated that the miR-200 family functions conversely to Snail to inhibit EMT and germ layer fate commitment in differentiating ESCs, and acts to maintain cells in an EpiSC-like stage. We show that progression past this stage occurs through the downregulation of the miR-200 family caused by Snail or by removal of activin, inducing EMT and skewing cell fates toward mesoderm or neuroectoderm, respectively. Together our data illustrate how Snail and the miR-200 family act in opposition to regulate EMT and exit from the epiblast state toward germ layer fate commitment.

Recent studies have reached different conclusions about whether miR-200 promotes [20] or attenuates differentiation [21]. While our gene chip analysis supports many of the indicated targets described in both reports, including Cadherin 11 [21], Neuropilin 1 [21], and Bmi1 [20], we do not see miR-200c/141 downregulation of Sox2 [20] (which is actually induced in our system). By using stable, inducible expression of the miR-200 family, a global transcriptional analysis, flow cytometry, and EpiSC culturing, we support the interpretation that the miR-200 family stalls ESC differentiation at a specific point in differentiation, the EpiSC stage.

Our demonstration that Snail biases fate determination builds on earlier studies of its role in the mouse embryo. Previously, it was found that Snail-deficient embryos die before E8.5 and arrest at the onset of gastrulation [7]. In Snail-deficient embryos, cells of the primitive streak fail to downregulate E-cadherin or migrate, and mesoderm formation is diminished as demonstrated by low expression of Brachyury. Interestingly, the epiblast and neuroectoderm marker *Otx2* fails to be restricted to the anterior segment in Snail-deficient embryos. Together with our findings, these data suggest that Snail plays a more active role in promoting differentiation and germ layer fate induction than previously considered. Given the expression of miR-200 in the early mouse embryo [36], including its restriction to nonmesoderm fates in the chick embryo [19], it is possible that Snail may also repress miR-200 in the early embryo to allow progression and differentiation of cells from the epiblast to early mesoderm.

In addition to mesoderm defects, the conditional deletion of Snail under control of the Meox2-Cre demonstrated failures in establishment of left-right asymmetry [8]. In this conditional Snail deletion, left-right axis formation is disrupted and nodal expression is no longer restricted to the left lateral plate mesoderm, perhaps suggesting redundant mechanisms between Snail's restriction of nodal in axis formation and Snail's restriction of activin/nodal in the maintenance of the epiblast. Snail and the miR-200 family both interact and influence multiple components of the TGFβ signaling pathway [15, 21, 37]. Thus, these factors could conceivably interact in a manner to antagonize one another's actions by opposing influences in the same signaling pathways, but this aspect of the molecular mechanism will require further study.

The basis for the molecular antagonism between Snail and activin signaling is not yet characterized, but previous studies have shown that another EMT transcription factor, Zeb2/SIP1, can antagonize activin-nodal signaling through its direct interaction with the MH2 domain of activated SMAD proteins [38]. Furthermore, Zeb2/SIP1 can promote neuroectoderm while inhibiting activin-induced mesendoderm induction in human ESCs [39]. Although the effect of Zeb2 on EMT in differentiating ESCs was not examined, Zeb2 was identified in a screen of transcription factors that were induced upon the inhibition of activin signaling. As we find that inhibiting activin signaling in differentiating ESC induces Zeb2 and EMT, we would hypothesize that Zeb2 may be responsible for the miR-200 repression mediating the EMT observed during later neuroectoderm differentiation, explaining why Zeb2-deficient mice display defects in cranial neural crest migration along with specification of neuroectoderm. Our data suggest a model in which Snail acts in concert with activin to promote mesoderm differentiation and migration, whereas Zeb2 functions in the absence of activin to promote neuroectoderm differentiation and delamination of neural crest (Fig. 7B). Both Snail and Zeb2 induction of EMT appear to oppose activin-induced maintenance of miR-200 and thereby promote progression past the epiblast state.

The relationship between Snail expression and miR-200 family members has recently been suggested [15, 40], but has not been examined in depth. One study indicated that over-expression of Snail in HCT116 cells could repress transcription from the regulatory regions of miR-200c and miR-141 [15]. A second study examined miRNAs regulated by Snail in MCF7 breast cancer cells and identified a number of miRNAs that were strongly induced by Snail [40]. This study also noted that miR-200 family members were repressed by Snail, but did not further examine the consequences of this repression functionally. Our study illustrates the first functional antagonism between the Snail and the miR-200 family and its consequential effects on EMT in a system relevant for gastrulation.

## CONCLUSION

We have demonstrated that Snail induces EMT in differentiating ESCs and promotes differentiation toward early mesoderm. Snail does this in part through the repression of miR-200, which cooperates with activin to maintain cells in a transcriptional state consistent with the ESD-EpiSC. Exit from the EpiSC-like state can be regulated through either the induction of Snail or the inhibition of activin, which generates an EMT concomitant with germ layer fate induction toward mesoderm or neuroectoderm, respectively.
