# Supplementary figures and images for: Snail and the microRNA-200 Family Act in Opposition to Regulate Epithelial-to-Mesenchymal Transition and Germ Layer Fate Restriction in Differentiating ESCs
Source: Stem Cells. 2011 Mar 10;29(5):764–76. doi: 10.1002/stem.628 (PMC3339404; doi:10.1002/stem.628)

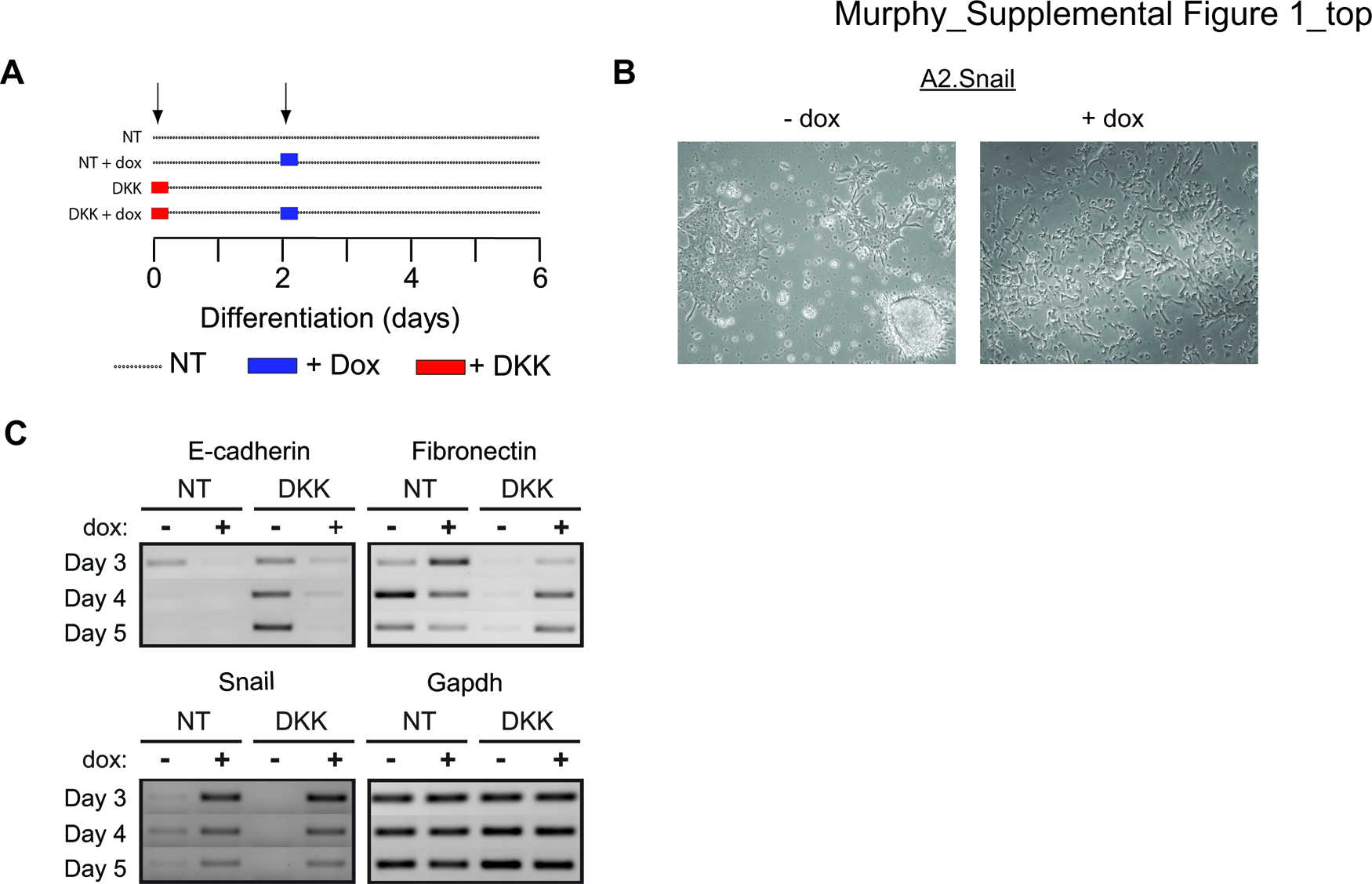

Supplement: Supplementary file 1 [file stem0029-0764-SD1.tif]
