# Supplementary material for: Snail and the microRNA-200 Family Act in Opposition to Regulate Epithelial-to-Mesenchymal Transition and Germ Layer Fate Restriction in Differentiating ESCs
Source: Stem Cells. 2011 Mar 10;29(5):764–76. doi: 10.1002/stem.628 (PMC3339404; doi:10.1002/stem.628)
Supplement: Supplementary file 10 [file stem0029-0764-SD10.doc]

**Supplemental Table 1.** Oligonucleotide primers used in this study.

|  | **CLONING PRIMERS** |
| --- | --- |
| **Name** | **Sequence** |
| C_mir200a/b/429 F (for A2.miR200a) | GCT**GGATCC**CTCCTTGGTTCCATGACCTGAGAA |
| C_mir200a/b/429 R (for A2.miR200a) | CTA**GATATC**GACTGGACCTGTTGTCTAGGCTATTCTG |
| C_mir200c/141 F (for A2.miR200c) | GCT**GGATCC**CCTTTCTTACGAAGACCGAGTCTCCA |
| C_mir200c/141 R (for A2.miR200c) | CTA**GATATC**CCTCAAGAGGAGGTGCCCAGG |
| C_mir335 F (for A2.miR335) | GAGAGAGTGGTGGGTCCAAGTAGGG |
| C_mir335 R (for A2.miR335) | GCAAGCTGACAGGACTTCAGGAGC |
| C_snail F | TAGGTCGCTCTGGCCAACATGC |
| C_snail R | AAGATGCCAGCGAGGATGGG |
|  |  |
|  | **q-RT-PCR PRIMERS** |
| FGF5 F | CACGAAGCCAGTGTGTTAAGTATTTTGG |
| FGF5 R | GCATCATCCAAAGCGAAACTTCAG |
| Fibronectin F | GGAATGGAAAGGGAGAATTCAAGTG |
| Fibronectin R | GGGGCAATTTACGTTAGTGTTTGTTC |
| Gapdh F | TGCCCCCATGTTTGTGATG |
| Gapdh R | TGTGGTCATGAGCCCTTCC |
| Nanog F | CTCTCCTCGCCCTTCCTCTGAAG |
| Nanog R | GGTGCTGAGCCCTTCTGAATCAG |
| N-cadherin F | ACAATCAACAATGAGACTGGGGACA |
| N-cadherin R | TCATTGACATCTGTCACCGTGATGA |
| Ncam1 F | TGCTGCGAACTAAGGATCTCATCTG |
| Ncam1 R | GCATTCTTGAACATGAGCTTCTGGA |
| Nodal F | GGATCATCTACCCCAAGCAGTACAATG |
| Nodal R | GCAAGCCAATTTCAGCACTCCC |
| Occludin F | CCTGGAGGTACTGGTCTCTACGTGG |
| Occludin R | TCTTTCCGCATAGTCAGATGGGG |
| Oct4 F | CAATGCCGTGAAGTTGGAGAAGG |
| Oct4 R | CGAAGCGACAGATGGTGGTCTG |
| Snail F | CGCTCTGAAGATGCACATCCGA |
| Snail R | TCACATCCGAGTGGGTTTGGAG |
| Sox2 F | CTCCATGACCAGCTCGCAG |
| Sox2 R | CCTTCTCCAGTTCGCAGTC |
| Zeb1 F | CCTACAGTCACTGCCCAGTTACCC |
| Zeb1 R | GCATACATTCCATTCTCTGTCTTCCG |
| Zeb2 F | CTGCCACTTTCATGCCACCAG |
| Zeb2 R | CGGAGTCTGTCATGTCATCTAGGC |
|  |  |
|  | **RT-PCR PRIMERS** |
| **Name** | **Sequence** |
| E-cadherin F | GTCCTGCCAATCCTGATGAAATTG |
| E-cadherin R | CACTGATATAATTATTCTGCATCTCCCA |
| Fibronectin F | GGAATGGAAAGGGAGAATTCAAGTG |
| Fibronectin R | GGGGCAATTTACGTTAGTGTTTGTTC |
| Gapdh F | TGCCCCCATGTTTGTGATG |
| Gapdh R | TGTGGTCATGAGCCCTTCC |
| Snail F | CGCTCTGAAGATGCACATCCGA |
| Snail R | TCACATCCGAGTGGGTTTGGAG |
