# Supplementary material for: Snail and the microRNA-200 Family Act in Opposition to Regulate Epithelial-to-Mesenchymal Transition and Germ Layer Fate Restriction in Differentiating ESCs
Source: Stem Cells. 2011 Mar 10;29(5):764–76. doi: 10.1002/stem.628 (PMC3339404; doi:10.1002/stem.628)
Supplement: Supplementary file 11 [file stem0029-0764-SD11.doc]

**Supplemental Table 2: MicroRNA Screen for Snail-Regulated MicroRNAs**

RNA samples were isolated on day 3 of ES cell differentiation, 24 hours after Snail induction

|  |  | DKK+  Snail |  | DKK |  | DKK+Snail/  DKK |
| --- | --- | --- | --- | --- | --- | --- |
| **Reporter Name** | **p-value** | **Mean** | **StDev** | **Mean** | **StDev** | **Fold Change** |
| mmu-miR-21 | 1.05E-08 | 61 | 7 | 678 | 87 | 0.09 |
| mmu-miR-295 | 4.56E-08 | 92 | 9 | 480 | 54 | 0.19 |
| mmu-let-7c | 5.72E-07 | 101 | 16 | 510 | 74 | 0.20 |
| mmu-miR-494 | 1.12E-06 | 1,073 | 29 | 1,687 | 73 | 0.64 |
| mmu-miR-292-3p | 1.23E-06 | 849 | 90 | 2,409 | 144 | 0.35 |
| mmu-miR-20b | 1.40E-06 | 1,983 | 83 | 3,768 | 237 | 0.53 |
| mmu-miR-106a | 2.65E-06 | 1,937 | 145 | 3,563 | 230 | 0.54 |
| mmu-miR-1187 | 2.99E-06 | 10,710 | 521 | 5,103 | 438 | 2.10 |
| mmu-miR-293 | 4.22E-06 | 5,990 | 377 | 10,014 | 356 | 0.60 |
| mmu-miR-669f | 5.60E-06 | 4,408 | 275 | 1,894 | 213 | 2.33 |
| mmu-miR-302b | 6.54E-06 | 1,056 | 260 | 175 | 38 | 6.02 |
| mmu-miR-574-5p | 6.64E-06 | 11,250 | 925 | 5,323 | 579 | 2.11 |
| mmu-miR-302d | 6.98E-06 | 429 | 75 | 117 | 12 | 3.66 |
| mmu-miR-690 | 7.01E-06 | 4,304 | 252 | 6,887 | 448 | 0.62 |
| mmu-miR-574-3p | 7.89E-06 | 6,013 | 610 | 2,328 | 338 | 2.58 |
| mmu-miR-720 | 7.96E-06 | 985 | 34 | 1,999 | 157 | 0.49 |
| mmu-miR-182 | 1.36E-05 | 558 | 55 | 1,009 | 72 | 0.55 |
| mmu-miR-714 | 2.26E-05 | 2,750 | 138 | 3,887 | 228 | 0.71 |
| mmu-miR-483 | 2.32E-05 | 682 | 55 | 296 | 38 | 2.30 |
| mmu-miR-294 | 3.18E-05 | 2,809 | 569 | 10,250 | 877 | 0.27 |
| mmu-miR-25 | 3.23E-05 | 827 | 55 | 1,349 | 132 | 0.61 |
| mmu-miR-466f-3p | 4.48E-05 | 8,627 | 681 | 3,981 | 553 | 2.17 |
| mmu-miR-466i | 4.88E-05 | 7,485 | 659 | 3,624 | 465 | 2.07 |
| mmu-miR-672 | 5.13E-05 | 890 | 108 | 444 | 60 | 2.01 |
| mmu-miR-23b | 6.30E-05 | 619 | 64 | 1,022 | 82 | 0.61 |
| mmu-miR-467f | 6.84E-05 | 8,071 | 602 | 4,282 | 547 | 1.89 |
| mmu-miR-466g | 7.11E-05 | 4,255 | 460 | 1,685 | 306 | 2.52 |
| mmu-miR-466f | 7.69E-05 | 1,472 | 210 | 647 | 111 | 2.27 |
| mmu-miR-466h | 8.80E-05 | 411 | 75 | 163 | 32 | 2.53 |
| mmu-miR-329 | 9.27E-05 | 1,188 | 98 | 1,788 | 141 | 0.66 |
| mmu-miR-17 | 9.49E-05 | 3,669 | 325 | 5,359 | 322 | 0.68 |
| mmu-miR-467a* | 1.10E-04 | 1,474 | 310 | 516 | 119 | 2.86 |
| mmu-miR-466j | 1.11E-04 | 824 | 159 | 309 | 63 | 2.66 |
| mmu-miR-1195 | 1.16E-04 | 631 | 37 | 457 | 33 | 1.38 |
| mmu-miR-106b | 1.22E-04 | 432 | 14 | 522 | 23 | 0.83 |
| mmu-miR-18a | 1.23E-04 | 311 | 14 | 505 | 43 | 0.62 |
| mmu-miR-20a | 1.25E-04 | 4,029 | 350 | 5,832 | 392 | 0.69 |
| mmu-miR-134 | 1.26E-04 | 1,676 | 156 | 1,107 | 88 | 1.51 |
| mmu-miR-23a | 1.34E-04 | 252 | 36 | 478 | 57 | 0.53 |
| mmu-miR-183 | 1.42E-04 | 1,578 | 135 | 2,251 | 135 | 0.70 |
| mmu-miR-191 | 1.51E-04 | 336 | 35 | 514 | 41 | 0.66 |
| mmu-let-7a | 2.08E-04 | 148 | 38 | 412 | 79 | 0.36 |
| mmu-miR-411* | 4.15E-04 | 814 | 104 | 1,270 | 134 | 0.64 |
| mmu-miR-290-5p | 4.22E-04 | 6,011 | 426 | 7,782 | 338 | 0.77 |
| mmu-miR-467b* | 4.59E-04 | 2,371 | 477 | 1,069 | 240 | 2.22 |
| mmu-miR-805 | 5.73E-04 | 2,120 | 179 | 1,469 | 166 | 1.44 |
| mmu-miR-467g | 5.82E-04 | 587 | 216 | 192 | 61 | 3.06 |
| mmu-miR-292-5p | 6.70E-04 | 2,395 | 215 | 3,623 | 106 | 0.66 |
| mmu-miR-19b | 7.91E-04 | 436 | 54 | 676 | 38 | 0.64 |
| mmu-miR-376b | 1.23E-03 | 1,869 | 112 | 1,414 | 125 | 1.32 |
| mmu-miR-680 | 1.64E-03 | 2,635 | 210 | 1,994 | 193 | 1.32 |
| mmu-miR-671-5p | 1.79E-03 | 874 | 32 | 1,047 | 68 | 0.83 |
| mmu-miR-379 | 2.44E-03 | 3,387 | 151 | 2,905 | 169 | 1.17 |
| mmu-miR-107 | 2.47E-03 | 627 | 28 | 553 | 23 | 1.13 |
| mmu-miR-689 | 2.81E-03 | 993 | 78 | 1,422 | 199 | 0.70 |
| mmu-miR-92a | 3.87E-03 | 2,916 | 325 | 3,919 | 412 | 0.74 |
| mmu-miR-16 | 3.99E-03 | 1,132 | 77 | 1,502 | 175 | 0.75 |
| mmu-miR-15b | 5.21E-03 | 1,585 | 119 | 1,952 | 175 | 0.81 |
| mmu-miR-130b | 6.52E-03 | 642 | 40 | 561 | 26 | 1.15 |

The following transcripts are statistically significant but have low signals (signal < 500)

|  |  | DKK+  Snail |  | DKK |  | DKK+Snail/  DKK |
| --- | --- | --- | --- | --- | --- | --- |
| **Reporter Name** | **p-value** | **Mean** | **StDev** | **Mean** | **StDev** | **Fold Change** |
| mmu-miR-27b | 1.58E-08 | 89 | 5 | 253 | 14 | 0.35 |
| mmu-miR-200c | 3.19E-06 | 91 | 14 | 301 | 36 | 0.30 |
| mmu-miR-674 | 4.22E-06 | 214 | 11 | 101 | 10 | 2.11 |
| mmu-miR-874 | 6.92E-06 | 46 | 5 | 176 | 31 | 0.26 |
| mmu-let-7b | 9.45E-06 | 78 | 17 | 357 | 68 | 0.22 |
| mmu-miR-200b | 9.62E-06 | 35 | 6 | 108 | 15 | 0.33 |
| mmu-miR-24 | 1.04E-05 | 104 | 10 | 249 | 10 | 0.42 |
| mmu-miR-466d-3p | 1.09E-05 | 133 | 23 | 43 | 6 | 3.12 |
| mmu-miR-489 | 1.86E-05 | 63 | 9 | 137 | 13 | 0.46 |
| mmu-let-7i | 2.32E-05 | 41 | 7 | 131 | 15 | 0.31 |
| mmu-let-7f | 2.47E-05 | 83 | 15 | 215 | 29 | 0.39 |
| mmu-miR-199a-3p | 3.47E-05 | 53 | 6 | 95 | 9 | 0.56 |
| mmu-miR-669c | 3.99E-05 | 137 | 25 | 43 | 10 | 3.17 |
| mmu-miR-712 | 4.49E-05 | 68 | 11 | 159 | 20 | 0.42 |
| mmu-miR-294* | 5.78E-05 | 60 | 6 | 104 | 7 | 0.57 |
| mmu-miR-291a-5p | 5.93E-05 | 106 | 15 | 218 | 17 | 0.49 |
| mmu-miR-27a | 7.37E-05 | 18 | 2 | 58 | 11 | 0.31 |
| mmu-miR-206 | 7.65E-05 | 77 | 11 | 31 | 6 | 2.50 |
| mmu-miR-877* | 8.97E-05 | 129 | 19 | 67 | 7 | 1.91 |
| mmu-miR-691 | 9.04E-05 | 136 | 15 | 72 | 10 | 1.91 |
| mmu-miR-297a | 1.03E-04 | 115 | 17 | 48 | 4 | 2.39 |
| mmu-miR-207 | 1.09E-04 | 138 | 8 | 71 | 9 | 1.93 |
| mmu-miR-669e | 1.30E-04 | 160 | 13 | 63 | 12 | 2.53 |
| mmu-miR-155 | 1.33E-04 | 36 | 6 | 76 | 12 | 0.48 |
| mmu-miR-466f-5p | 1.39E-04 | 164 | 30 | 67 | 13 | 2.45 |
| mmu-miR-669h-3p | 1.46E-04 | 265 | 84 | 71 | 22 | 3.74 |
| mmu-miR-188-5p | 2.38E-04 | 120 | 9 | 35 | 6 | 3.38 |
| mmu-miR-322* | 2.80E-04 | 48 | 11 | 17 | 4 | 2.88 |
| mmu-miR-15a* | 2.86E-04 | 143 | 16 | 66 | 12 | 2.16 |
| mmu-miR-696 | 2.91E-04 | 150 | 3 | 77 | 9 | 1.95 |
| mmu-miR-214 | 3.33E-04 | 218 | 20 | 154 | 12 | 1.42 |
| mmu-miR-466a-3p | 3.37E-04 | 74 | 14 | 23 | 7 | 3.16 |
| mmu-miR-363 | 4.04E-04 | 109 | 22 | 225 | 35 | 0.48 |
| mmu-miR-291a-3p | 4.16E-04 | 21 | 6 | 78 | 4 | 0.27 |
| mmu-miR-125b-5p | 4.33E-04 | 133 | 13 | 194 | 18 | 0.69 |
| mmu-miR-351 | 4.40E-04 | 124 | 6 | 38 | 10 | 3.24 |
| mmu-miR-468 | 5.38E-04 | 170 | 38 | 59 | 18 | 2.85 |
| mmu-miR-652 | 5.65E-04 | 61 | 7 | 35 | 5 | 1.76 |
| mmu-miR-7a | 5.92E-04 | 136 | 26 | 283 | 56 | 0.48 |
| mmu-miR-327 | 5.98E-04 | 100 | 12 | 62 | 4 | 1.62 |
| mmu-miR-29a | 6.46E-04 | 45 | 14 | 139 | 24 | 0.32 |
| mmu-miR-346 | 7.19E-04 | 419 | 57 | 234 | 42 | 1.79 |
| mmu-miR-143 | 8.35E-04 | 25 | 5 | 46 | 7 | 0.53 |
| mmu-miR-195 | 9.28E-04 | 172 | 14 | 118 | 15 | 1.46 |
| mmu-miR-411 | 9.85E-04 | 121 | 13 | 168 | 10 | 0.72 |
| mmu-let-7d | 1.18E-03 | 74 | 16 | 155 | 33 | 0.48 |
| mmu-miR-467e* | 1.22E-03 | 178 | 58 | 56 | 27 | 3.20 |
| mmu-miR-299* | 1.26E-03 | 131 | 13 | 174 | 13 | 0.75 |
| mmu-miR-669d | 1.26E-03 | 102 | 22 | 39 | 11 | 2.61 |
| mmu-miR-374 | 1.27E-03 | 119 | 22 | 226 | 44 | 0.52 |
| mmu-miR-708 | 1.32E-03 | 57 | 5 | 79 | 8 | 0.72 |
| mmu-miR-712* | 1.41E-03 | 54 | 3 | 106 | 17 | 0.51 |
| mmu-miR-466b-3-3p | 1.55E-03 | 68 | 17 | 27 | 7 | 2.52 |
| mmu-miR-31 | 2.30E-03 | 40 | 5 | 55 | 5 | 0.74 |
| mmu-miR-183* | 2.41E-03 | 53 | 4 | 77 | 11 | 0.69 |
| mmu-miR-301a | 2.45E-03 | 43 | 5 | 61 | 8 | 0.71 |
| mmu-miR-485 | 2.46E-03 | 114 | 9 | 84 | 10 | 1.35 |
| mmu-miR-429 | 2.52E-03 | 16 | 5 | 34 | 9 | 0.46 |
| mmu-miR-221 | 2.58E-03 | 55 | 7 | 75 | 7 | 0.73 |
| mmu-miR-150* | 2.75E-03 | 84 | 5 | 46 | 10 | 1.82 |
| mmu-miR-34c* | 2.78E-03 | 96 | 14 | 59 | 11 | 1.63 |
| mmu-miR-342-5p | 3.27E-03 | 87 | 6 | 48 | 10 | 1.82 |
| mmu-miR-713 | 3.67E-03 | 62 | 10 | 32 | 8 | 1.93 |
| mmu-miR-501-3p | 4.38E-03 | 39 | 8 | 23 | 2 | 1.65 |
| mmu-miR-18b | 4.43E-03 | 33 | 8 | 66 | 6 | 0.50 |
| mmu-miR-295* | 4.79E-03 | 53 | 7 | 78 | 13 | 0.68 |
| mmu-miR-674* | 5.06E-03 | 43 | 7 | 30 | 3 | 1.44 |
| mmu-miR-328 | 5.29E-03 | 64 | 10 | 44 | 3 | 1.45 |
| mmu-let-7g | 6.75E-03 | 27 | 6 | 48 | 10 | 0.55 |
| mmu-miR-19a | 6.76E-03 | 14 | 3 | 24 | 6 | 0.58 |
| mmu-let-7e | 7.17E-03 | 324 | 56 | 225 | 30 | 1.44 |
| mmu-miR-770-3p | 8.17E-03 | 109 | 15 | 79 | 10 | 1.37 |
| mmu-miR-467h | 8.93E-03 | 61 | 23 | 24 | 8 | 2.55 |
| mmu-miR-125a-5p | 9.72E-03 | 345 | 42 | 236 | 47 | 1.46 |
